# Revalidation of Proactive Gastrostomy Tube Placement Guidelines for Head and Neck Cancer Patients Receiving Helical Intensity-Modulated Radiotherapy †

**DOI:** 10.3390/curroncol31110512

**Published:** 2024-11-06

**Authors:** Teresa E. Brown, Angela Byrnes, Aaron C. Chan, Kathleen Dwyer, Anna Edwards, Claire L. Blake, Merrilyn D. Banks, Brett G. M. Hughes, Charles Y. Lin, Lizbeth M. Kenny, Ann-Louise Spurgin, Judith D. Bauer

**Affiliations:** 1 Dietetics & Food Services, Royal Brisbane & Women’s Hospital, Brisbane, QLD 4029, Australia; 2 School of Human Movement & Nutrition Sciences, University of Queensland, Brisbane, QLD 4072, Australia; 3 Nutrition & Food Services, Ipswich Hospital, Ipswich, QLD 4305, Australia; 4 Nutrition & Dietetics, Toowoomba Hospital, Darling Downs Health, Toowoomba, QLD 4350, Australia; 5 Cancer Care Services, Royal Brisbane & Women’s Hospital, Brisbane, QLD 4029, Australia; 6 School of Medicine, University of Queensland, Brisbane, QLD 4072, Australia; 7 Department of Speech Pathology, Royal Brisbane & Women’s Hospital, Brisbane, QLD 4029, Australia; 8 Department of Nutrition, Dietetics & Food, Monash University, Melbourne, VIC 3800, Australia

**Keywords:** head and neck cancer, radiotherapy, gastrostomy, enteral feeding, nutrition

## Abstract

The Royal Brisbane and Women’s Hospital (RBWH) Swallowing and Nutrition Management Guidelines for Patients with Head and Neck Cancer were developed to enable evidence-based decision-making by the Head and Neck Multidisciplinary Team (H&N MDT) regarding enteral nutrition support options. The purpose of this study was to revalidate these guidelines in a cohort of patients receiving helical intensity-modulated radiotherapy (H-IMRT) compared to a historical cohort who received primarily 3D-conformal radiotherapy. Eligible patients attending the RBWH H&N MDT between 2013 and 2014 (n = 315) were assessed by the guidelines, with high-risk patients being recommended proactive gastrostomy tube placement. Data were collected on guideline adherence, gastrostomy tube insertions, the duration of enteral tube use and weight change. Sensitivity, specificity and positive predictive and negative predictive values were calculated and compared with the historical cohort. Overall guideline adherence was 84%, with 60% and 96% adherence to the high-risk and low-risk pathways, respectively. Seventy patients underwent proactive gastrostomy tube placement (n = 62 high-risk; n = 8 low-risk). Validation outcomes were sensitivity 73% (compared to 72%) and specificity 86% (compared to 96%). The guidelines yielded a high sensitivity and specificity, remaining valid in a cohort of patients treated with H-IMRT. Further studies are recommended to improve the sensitivity and understand the decrease in specificity in order to make ongoing guideline improvements.

## 1. Introduction

Head and neck cancers (HNC) are a heterogeneous group of malignancies including the oral cavity, oropharynx, nasopharynx, hypopharynx and larynx, as well as the skin, paranasal sinuses and salivary glands [1]. Historically, tobacco and alcohol were the primary risk factors for developing HNC, but human papilloma virus (HPV) infection is also an established risk factor for oropharyngeal cancers [1]. A recent systematic review has demonstrated that the global incidence of HPV-related HNC subsites has risen, while most of the HPV-unrelated subsites declined or remained stable [2]. Cancers of the head and neck are treated by local resection, radiation treatment and/or by systemic therapies such as chemotherapy or immunotherapy, with many patients undergoing multi-modality treatment.

Patients with HNC are at a high-risk of malnutrition due to multiple reasons, including tumor obstruction impacting oral intake, oropharyngeal dysphagia or other side effects of their treatment [3,4]. Malnutrition in patients with HNC is adversely associated with treatment tolerance and outcomes, wound healing, morbidity, mortality, quality of life and survival [5], and thus management through nutrition intervention is essential to optimize patient outcomes [6]. Depending on tumor site, extent and the treatment given, patients can lose a significant amount of weight or may be unable to eat and drink, subsequently requiring artificial nutrition and hydration support in the form of a nasogastric or gastrostomy tube [7]. Feeding tubes may be inserted ‘proactively’ (prior to commencing treatment), or ‘reactively’ (as required throughout treatment). The timing of feeding tube placement remains controversial in the literature [8] and there is large variation internationally in dietetic and oncological practice regarding enteral nutrition management [9,10,11,12].

The identification of patients who are at high nutritional risk with the proactive placement of feeding tubes and initiation of early tube feeding has been shown to improve adherence to enteral nutrition recommendations and reduce client distress regarding tube feeding, thus helping to prevent nutritional decline [13,14]. Proactive tube placement is known to help reduce potential weight loss, may reduce unplanned hospital admissions, and may improve quality of life during and post-treatment [15,16,17,18]. Proactive gastrostomy use has also been associated with a shorter length of hospital stay [19] and reduced weight loss at 6 weeks post-treatment [20,21]. As such, proactive tube placement is often seen as the preferred method when compared to reactive placement by many clinicians. Alternatively, the use of reactive enteral nutrition, where recommendations for a gastrostomy or nasogastric tube placement is initiated once a nutritional decline develops [22], is advocated by some facilities. Proponents of reactive enteral nutrition often rationalize the practice given the belief that this may reduce tube dependency [23]; however, numerous studies have demonstrated that proactive feeding tube placement does not increase long-term reliance on tube use [4,19,24,25]. Other benefits of reactive feeding tube use include a shorter duration of nutrition support [26] and lower healthcare costs for the placement of a reactive nasogastric tube [20]. On the other hand, disadvantages to a reactive nasogastric tube include the impact of having an altered body image, limitations in social activities, the perception of inconvenience and more frequent tube dislodgements [20].

The Royal Brisbane & Women’s Hospital (RBWH) Swallowing and Nutrition Management Guidelines for Patients with Head and Neck Cancer (herein referred to as “the guidelines”) were developed and implemented in 2006 and first validated in 2013 [27]. The guidelines help to identify patients who are at high nutritional risk, including those for whom it is anticipated that enteral feeding will be required for longer than 4 weeks, supporting the RBWH Head and Neck Cancer Multidisciplinary Team (H&N MDT) in decisions regarding proactive feeding tube placement. The guidelines also assist in determining which patients may benefit from receiving reactive enteral nutrition once treatment has commenced and oral intake is <60% of requirements for an anticipated >10 days. Based on international guidelines, a nasogastric tube is recommended if the duration of feeding is anticipated to be <4 weeks, and a reactive gastrostomy tube is recommended if feeding is likely to be required for >4 weeks [6,28].

Helical intensity-modulated radiotherapy (H-IMRT), a new generation of rotational IMRT, was introduced to RBWH in 2010. IMRT techniques are the current gold-standard for treating cancers of the head and neck (compared to traditional 3D-conformal radiotherapy) due to the ability to deliver a more precise dose of radiation where needed whilst sparing surrounding critically important structures [29]. In theory, by preserving organs and tissues involved in swallowing, H-IMRT is proposed to lessen the radiation-induced nutrition impact symptoms and risk of malnutrition, and thus potentially reduce the risk of requiring feeding tube placement. This reduction in toxicity was supported by Ghosh et al., who demonstrated a reduction in Grade 3 toxicities (xerostomia, mucositis, dysphagia) and thus feeding tube use in patients treated with IMRT versus 3D-conformal radiotherapy [30]. Similarly, only 47% of patients treated with parotid-sparing IMRT in a UK study required a feeding tube, and although swallowing toxicity was still present in the acute phase, long-term improvement was seen at 12 months [31]. Additional local data collected on toxicities during H-IMRT also implied that patients remained at risk of acute toxicities during treatment, with 38% requiring full/partial enteral nutrition support, but the majority of toxicities had returned to baseline by 3 months post-treatment [32]. Therefore, although the toxicity profiles of this new treatment have improved and achieve better long-term functional outcomes, patients are still impacted by acute toxicities during treatment and may require tube feeding [33]. Prompted by the introduction of H-IMRT and the possible reduced need for tube feeding, the guidelines were revalidated in 2016 using a mixed cohort from 2010 to 2011 [34] with 28% of patients receiving H-IMRT and the remainder 3D-conformal radiotherapy. With high specificity (96%) and sensitivity (72%), the study confirmed that the guidelines remained valid for patients treated with H-IMRT; however, the study was limited by the small sample size of patients who received H-IMRT.

As most patients with HNC at the RBWH are now treated with H-IMRT, it is important to ensure the guidelines remain valid as oncology treatments evolve. In this work, the study presented in [35] is expanded upon with the aim to revalidate the guidelines with a larger cohort of patients who received H-IMRT, compared to the historical cohort primarily receiving 3D-conformal radiotherapy, and to identify any other factors that might help predict the need for proactive feeding tube placement. The results of this study will then provide further information to guide clinical decision-making on appropriate enteral feeding tube placement in the contemporary management of patients with HNC.

## 2. Materials and Methods

### 2.1. Study Population

Patients were eligible for this retrospective cohort study if they attended the RBWH for assessment and completed curative intent treatment of a diagnosed mucosal HNC between July 2013 and June 2014. Patients were deemed ineligible if they were only admitted to the short-stay surgical unit, as they were not referred to the hospital dietitian or speech pathologist. Patients were further excluded from analysis if there were incomplete data (no end-point weight or lost to follow-up), they declined dietetic services or had a feeding gastrostomy tube in situ at baseline. The latter were excluded because the primary intent of this study was to determine if the guidelines can predict the need for gastrostomy with high sensitivity and specificity, and this decision-making would not be required in these instances where a tube was already in place. This study was assessed by the RBWH Human Research Ethics Committee (HREC/13/QRBW/20) and it was confirmed that it met the National Health and Medical Research Council guidance for Quality Assurance and Evaluation Activities; furthermore, it was exempt from full ethical review.

### 2.2. Study Design, Data Collection and Outcomes

The study design and data collection methods for this study were the same as those used for the previous validation studies by Brown et al. [27,34], including the use of the Seventh Edition of the American Joint Committee on Cancer (AJCC) Cancer Staging Manual for TNM staging. Key outcomes for this study include adherence to the guidelines and any changes in sensitivity and specificity, or positive and negative predictive values, compared to the 2013 validation. Due to the limitations identified in the previous validation studies, the final definition used to confirm appropriateness for proactive tube placement was changed; in this study, patients were confirmed as high-risk if they used a feeding tube (proactive or reactive) to meet any proportion of nutritional requirements for >4 weeks or had lost a significant amount of weight by treatment end (≥10% weight loss based on baseline body weight pre-treatment at the H&N MDT). The determination of guideline adherence by the H&N MDT was collected prospectively and determined by confirming whether the correct intervention had been applied following risk classification. So, adherence was confirmed if patients classified as high-risk received the recommended proactive gastrostomy, and if patients classified as low-risk did not receive a proactive gastrostomy. Data on complications related to gastrostomy placement were collected for the 30-day post-insertion period, with a ‘major’ complication defined as requiring admission for intravenous antibiotics, surgical intervention or blood transfusion.

### 2.3. Statistical Analysis

Descriptive statistics were used to report patient demographics and clinical characteristics. Numerical variables were reported as mean and standard deviation and categorical variables were reported as percentage frequency. Differences between cohorts were compared using the *t*-test for numerical variables and the chi-squared test for categorical variables, with *p* < 0.05 set for statistical significance. Logistic regression was used to determine univariate associations between patient characteristics available at the H&N MDT and the primary outcome of need for proactive gastrostomy, with the *p*-value of the Wald statistic reported in the results. Variables with *p* < 0.1 in univariate analysis were entered into the multivariate model using backward logistic regression to assess the effects of gender, malnutrition risk (as per the malnutrition screening tool [MST]), tumor site, treatment modality, and tumor stage on the need for a proactive gastrostomy. Variables were selected with this higher *p*-value cut-off of <0.1 to avoid omitted variable bias, as otherwise important predictors may be omitted when their association only becomes apparent when other variables are taken into account. Data were analyzed using IBM SPSS Statistics for Windows version 21.0 (Armonk, NY, USA: IBM Corp.).

## 3. Results

### 3.1. Patient Characteristics

**Figure 1:**
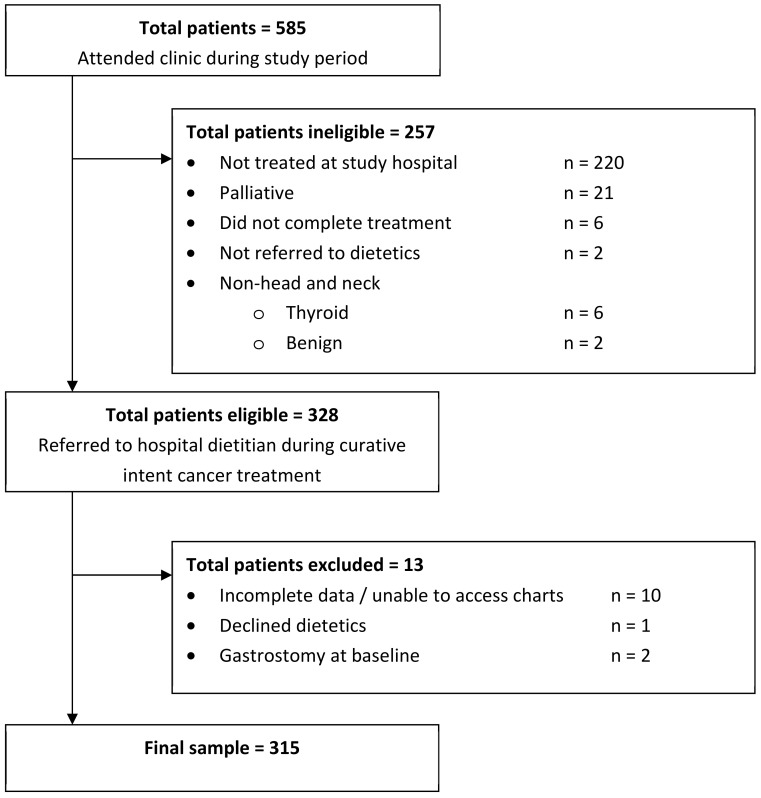
CONSORT diagram to illustrate eligible patient sample with inclusion and exclusion criteria.

During the one-year study period, 585 patients attended the hospital for assessment for HNC, of which 257 patients were ineligible as they were not referred to the hospital dietitian during curative intent treatment, and a further 13 patients were excluded (10 due to missing data). After the inclusion and exclusion criteria were applied, the final sample size was 315 patients (Figure 1). Only two patients had undergone prior gastrostomy in situ at baseline. Patient characteristics are summarized in Table 1.

There were no differences between the current and historical cohorts, except for N stage (*p* = 0.006), with the current study cohort having higher rates of N0 disease (40% vs. 34%) and N2 disease (40% vs. 33%) and lower rates of N1 disease (11% vs. 17%) and recurrence (7% vs. 14%). In the current cohort, most patients were male (76%), and the median age was 65 years (range 28–92 years). Half the population (51%) had a lesion of the oral cavity or oropharynx, and T stage classification was distributed evenly. Most patients received radiotherapy as part of their treatment (81%). There were 193 patients who received H-IMRT, accounting for 76% of patients treated with radiotherapy, compared to only 33% in the historical cohort (*p* < 0.001).

### 3.2. Adherence to the Guidelines

Overall adherence to the guideline was 84%. Adherence to the high-risk pathway was 60%; that is, 62 out of 104 patients that were deemed to be high-risk underwent proactive gastrostomy tube placement. Adherence to the low-risk pathway was 96%, reflecting a total of eight patients who underwent proactive gastrostomy tube placement despite being considered low-risk.

### 3.3. Tube Feeding and Weight Change Outcomes

**Figure 2:**
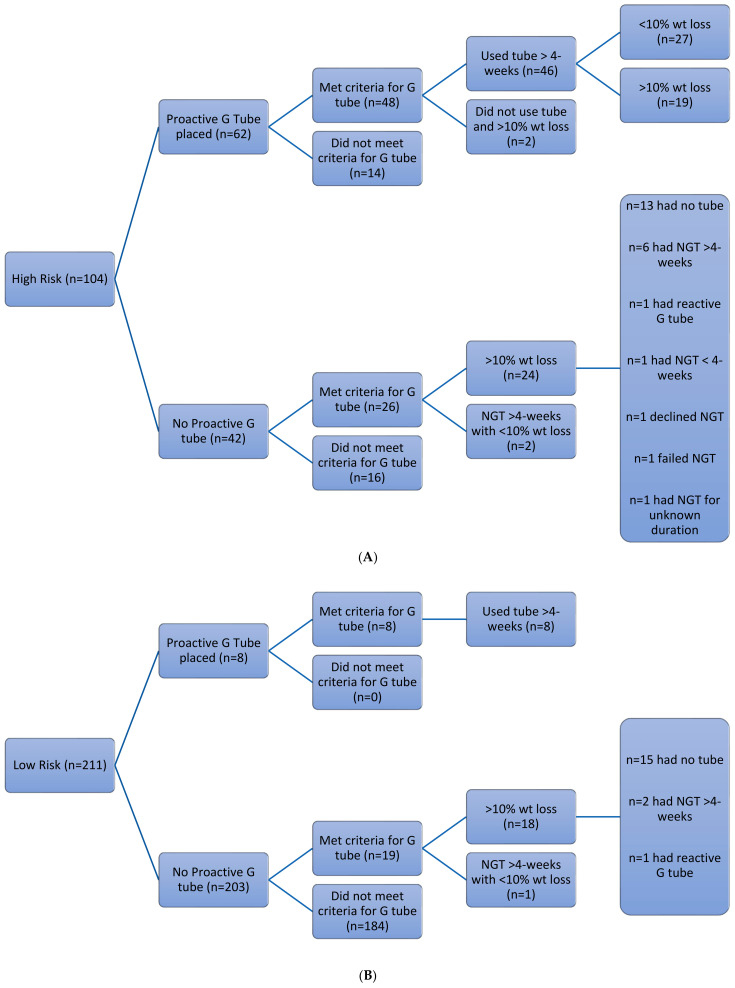
(**A**): Outcomes of the high-risk patient group (n = 104). (**B**): Outcomes of the low-risk patient group (n = 211).

Of the 62 high-risk patients in whom a proactive gastrostomy was placed, 48 patients (77%) met the final criteria for gastrostomy placement. Forty-six required a feeding tube to meet any proportion of nutritional requirements for >4 weeks and two patients did not use their tube for >4 weeks (reasons not recorded), subsequently losing >10% body weight. The remaining 42 high-risk patients did not undergo proactive gastrostomy, with the reasons for this including the following: consultant decision = 20; patient decision = 10; contraindication = 2; procedure canceled = 1; or unknown = 9. A total of 26 of these 42 patients (62%) met the final criteria for a proactive tube, with their actual tube feeding and weight loss outcomes shown in Figure 2A.

Of the 211 patients classified as low-risk, eight patients underwent gastrostomy tube placement for the following reasons: predicted post-op requirement (n = 4); consultant decision (n = 3); risk downgraded to low after tube placed (n = 1). All patients used their tube for >4 weeks and had <10% weight loss. Of the remaining 203 low-risk patients, a further 19 patients met the final high-risk criteria (see Figure 2B). Therefore, of the patients categorized as low-risk by the guidelines, a total of 27 (13%) met the final outcome criteria for needing proactive gastrostomy insertion.

### 3.4. Gastrostomy Complications

Gastrostomy tubes were placed in 70 patients overall, with a ‘major’ complication rate of 2.9% (n = 2). The major complications reported at one month post-insertion were one gastrostomy site infection, requiring IV antibiotics, and one abscess that required surgical drainage and IV antibiotics.

### 3.5. Validation of the Guideline

Of the 315 patients included in the study, 104 patients initially met the predefined criteria for requiring proactive gastrostomy tube placement. When retrospectively reviewing patient outcomes, 74 of these patients met the final criteria for proactive gastrostomy tube placement, giving a positive predictive value of 71%. Of the 211 patients who were classified by the guidelines as low-risk and deemed not to require a proactive gastrostomy, 27 patients met the final outcome criteria, giving a negative predictive value of 87%. The guideline sensitivity was 73% and specificity was 86% (Table 2); these values are compared to previous validation studies (Table 3).

### 3.6. Proactive Gastrostomy Predictors

A further investigation of the false positive cases (n = 30) was completed in order to determine any similarities (Table 4). Two patients were deemed high-risk due to severe malnutrition on presentation, although they did not undergo proactive tube placement. They were treated with surgery alone and gained weight post-operatively.

The remaining 28 patients received either definitive or adjuvant chemoradiotherapy. Most of these patients had oropharyngeal tumors (n = 20, 67%), with the majority being smaller in size: T0/Tx (n = 4), T1 (n = 4), and T2 (n = 14); these tumors accounted for 74% of this sample. Of these 30 patients, 14 patients underwent proactive gastrostomy placement, and in retrospect, this was not required.

A logistic regression analysis was performed to assess the effects of anticipated predictors on the need for a proactive gastrostomy (Table 5). The logistic regression model was statistically significant (χ^2^(9) = 125.51, *p* < 0.001), explained 50% (Nagelkerke R2) of the variance in need for proactive gastrostomy and correctly classified 79.7% of cases. Sex, age and malnutrition risk were not statistically significant and therefore not included in the final model. The tumor site, T stage and treatment modality were all independent predictors on multivariable analysis (*p* = 0.017, *p* = 0.004 and *p* < 0.001, respectively).

## 4. Discussion

This study has demonstrated that the guidelines have remained valid over time, following the uptake to predominantly H-IMRT treatment at our institution, with a high specificity from 96% to 86%, a positive predictive value from 92% to 71% and negative predictive value from 82% to 87%, and a moderate sensitivity of 72–73%. Overall, 32% (n = 101) of this patient cohort met the criteria for requiring a proactive gastrostomy tube, which is still a high proportion of patients requiring enteral nutrition support, and so the guidelines remain valid for use in clinical practice in settings with newer radiotherapy techniques to help accurately identify this sub-group of higher risk patients. The slight change to validation results may also be attributed to the change in the definition used to confirm appropriateness for proactive tube placement compared to previous studies. The decline in specificity is due to more false positives, meaning that the guidelines are potentially over-predicting the number of patients that require a tube. Given the differences in the cohort being largely driven by the higher proportion treated with H-IMRT, it is hypothesized that this change is attributable to this improved targeted form of treatment. At the same time, the adherence rate to the guidelines declined to 84%, which is lower than in previous validation studies (93% 2010–11; 89% 2007–08 cohorts) [27,34]. This may reflect changing clinician attitudes towards less of a perceived patient risk from treatment with H-IMRT compared to 3D-conformal radiotherapy.

Various studies report reduced treatment-related toxicities with H-IMRT, particularly in relation to salivary preservation, xerostomia, dysphagia and late treatment toxicities [33,36,37,38]. This is likely due to the reduced toxic effects from H-IMRT of sparing normal musculature while preserving locoregional control of malignancies [39]. A large retrospective study (n = 2993) also demonstrated a shorter duration of feeding tube placement in patients treated with IMRT vs. 3D-conformal radiotherapy [40]. However, this current study suggests patients treated for HNC that undergo H-IMRT still demonstrate the same level of acute nutritional risk as those undergoing 3D-CRT, despite the perceived lower risks from the improved dosimetry distribution and clinical efficacy of H-IMRT. In this current study, 101/315 patients met the outcome criteria of needing a proactive gastrostomy (i.e., they required tube feeding for >4 weeks and/or had >10% weight loss), with 74 of these correctly classified by the guidelines as high-risk and 27 misclassified as low-risk. Likewise, in a previous study by Brown et al. [41], the need for proactive gastrostomy (n = 49, 92% vs. n = 115, 86%, *p* = 0.213), median % weight change (−7.2% vs. −7.3%, *p* = 0.573) and severe weight loss incidence (28% vs. 27%, *p* = 0.843) were not significantly different between patients receiving H-IMRT vs. 3D-CRT. A recent study by Wang et al. [42] of seventy-nine patients with esophageal cancer showed that they also did not have a reduced risk of malnutrition when treated with H-IMRT compared to 3D-CRT, suggesting that the risk of acute nutritional decline remains high, despite radiotherapy techniques for various oncological populations.

The decision regarding tube feeding and proactive versus reactive placement remains a contentious issue in current clinical practice, with pros and cons to both approaches reported in a recent systematic review [7]. There are concerns that proactive gastrostomy may be associated with reduced swallow function and long-term tube dependency [9,43,44]. However, Dechaphunkul, et al. have reported that the partial use of a feeding tube alongside oral intake is tolerated, has optimal long-term outcomes with the maintenance of weight and nutritional status and a reduced risk of tube dependence compared to those that rely fully on tube feeding [44]. In addition, a number of studies report that proactive gastrostomy placement is associated with increased completion rates for chemotherapy [45,46,47,48], which can lead to improved survival outcomes [48,49], and lower hospitalization rates [50].

The results from this study suggested that the guidelines required refinement to improve their diagnostic accuracy. In our study, it was observed that 14 patients were deemed to undergo proactive gastrostomy unnecessarily. This sample size was too small to undertake further statistical modeling, but a further analysis of their nutrition outcomes was undertaken. This observation of unnecessary placement was based on the definition of either not using their feeding tube (or using for <4 weeks) and achieving <10% weight loss. However, in some cases, not using the tube was a patient decision, despite recommendations from the healthcare team, and multiple reasons for patient non-adherence have been reported in the literature [51]. In review of this group, the median weight loss was 6%, with 8/14 patients losing >5%. This degree of weight loss is still deemed critical and associated with poorer survival outcomes [52], and thus more intensive nutrition intervention could have been beneficial.

**Figure 3:**
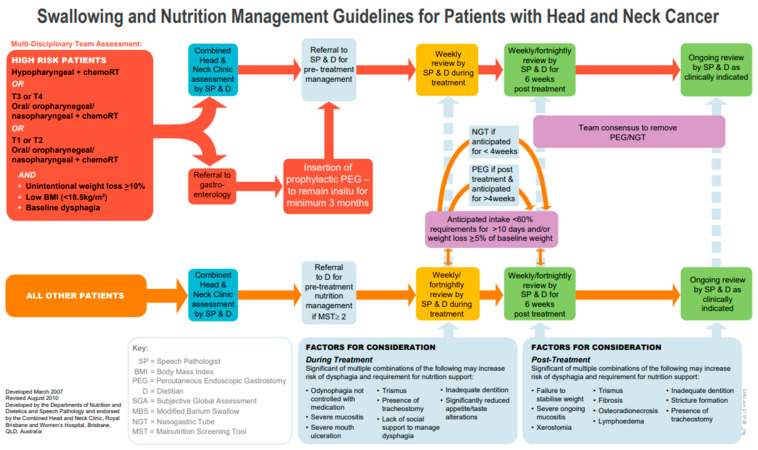
RBWH Swallowing and Nutrition Management Guidelines for Patients with Head and Neck Cancer with updated high-risk categories (revised 2021).

Secondly, there were 30 false positives whose clinical characteristics were described in Table 4 and had predominantly smaller T stages (T0–T2, n = 22, 73%), oropharyngeal cancers (n = 20, 67%), or were undergoing definitive or adjuvant chemoradiotherapy (n = 28, 93%). The key variables to predict the need for proactive gastrostomy identified in our study in Table 5 included tumor site, T stage and treatment. The current literature continues to support the idea that the tumor site has a role in determining the need for tube feeding or the risk of malnutrition or critical weight loss, with oral cavities [49,53,54], the oropharynx [46,49,53,54,55,56], the nasopharynx [17], the hypopharynx [17,46,57] and the supraglottis [46,53,54], all important locations. Similar to our findings, the T stage has also been found to be an important predictor of tube feeding in other studies [23,53,54,58] and some studies have also reported N staging to be important [17,46,59,60], although this was not confirmed by our study on multivariable analysis. A higher N stage (N2/N3) was associated with the need for proactive gastrostomy on univariate analysis (*p* < 0.001), but as N staging is associated with how treatment is determined, when added to the model, it was no longer significant. Finally, concurrent chemoradiotherapy treatment remains an important predictor of feeding tube use, malnutrition risk and critical weight loss [17,46,55,56,59,61,62,63]. The literature also reports that baseline dysphagia [59,60,64,65] and malnutrition risk, low BMI or pre-treatment weight loss [23,43,47,65,66] are related to an increased risk for a gastrostomy. Based on the findings from the literature and the findings from our current study, some minor adjustments were made to the guidelines’ high-risk category to incorporate T staging for some tumor subsites and to account for baseline nutrition or swallow deficits for those with T1/T2 disease, along with the removal of severe malnutrition as a stand-alone high-risk indicator (see Figure 3).

Our study did not identify gender as a factor in predicting the need for proactive gastrostomy, and whilst age was significant in univariate analysis (*p* = 0.002), it was not significant in the multivariable analysis. Only one other older study has reported an association of the female gender with feeding tube placement [67]. Several studies have reported age being associated with an increased risk of feeding tube placement [40,68,69]. More recently, one systematic review concluded that increasing age was associated with an increased risk of reactive feeding tube placement [15] and another reported that increasing age was associated with prolonged feeding tube dependency [43], suggesting that older patients may require closer monitoring during treatment and more support post-treatment.

The only other published guidelines that have been developed and validated to predict proactive gastrostomy placement are by Willemsen et al. (2022) [65]. Similar to our guidelines, they are based on the same prediction outcome of requiring tube feeding for >4 weeks, which is the standard recognized by international guidelines where gastrotomy insertion is recommended when the duration of feeding tube use is expected to exceed 4 weeks [6,28]. However, the outcome of weight loss without the presence of a feeding tube has not been considered in this case. The clinical variables used in their model are similar to this study with respect to tumor site and treatment with chemoradiotherapy, but differ in other aspects, with the inclusion of pre-treatment weight loss, texture modified diet at baseline, performance status, N stage and mean radiotherapy dose to the parotid gland and oral cavity. The positive predictive values are reasonably similar across both models, with our current study at 71% compared to 81% [65], and with our model at a higher negative predictive value at 87% compared to 42% [65].

While a high negative predictive value is important, the specificity has more clinical relevance in avoiding unnecessary gastrostomy tube placements and potential complications. The systematic review and meta-analysis by Grant et al., in an exclusive HNC population undergoing endoscopic gastrostomy placement, reported a procedure-related mortality rate of 2.2% and a major complication rate of 7.4% [70]. Our current study cohort compares favorably with a low rate of major complications at 2.9% (n = 2 patients) and nil mortality associated with tube placement. Given the small numbers affected, it was not possible to analyze factors that may have contributed to these two occasions whereby IV antibiotics were indicated to treat the infection and abscesses. However, patients with HNC are known to have a higher risk of infections from gastrostomy placement compared to non-HNC patients [71], which is attributable to their immunocompromised status during chemotherapy, and thus the timing of tube placement (proactive versus reactive) may also be a factor in mitigating risk. Kucha et al. described their complication rates, comparing patients who had the tube placed prophylactically prior to treatment (n = 445, 45%) to those that had it placed during or after treatment (n = 544, 55%), and found that the latter group had a three-fold higher risk of major adverse events/complications, therefore recommending that tube placement is undertaken prior to treatment [72]. The placement technique is another factor to consider. Several studies have demonstrated higher rates of major complications [70], any complications generally [73] and tube dislodgements [72] in radiologically inserted tubes compared to endoscopic placement.

The limitations of our guidelines are that they have not been externally validated. A strength of our guidelines is that all clinical information to determine risk category is readily available for use at the tumor board meetings, which improves the ease and success of implementation into practice. It should also be considered that the treatment paradigm for oropharyngeal cancer has somewhat changed in the last few years due to the advent of transoral robotic surgery (TORS) [74]. At our hospital, some patients with early-stage oropharyngeal cancer (T1/T2) are now being treated with transoral robotic resection and there is yet to be a harmonized approach on the optimal adjuvant treatment. In addition, more patients with lateralized oropharyngeal cancer are now being offered unilateral rather than bilateral radiotherapy, which may also impact on the requirement of tube feeding. The TNM staging system has also been updated to the eighth edition to account for the HPV-related oropharyngeal cancers which have a more favorable prognosis [75], which would also alter the N staging in this study and potentially influence the results. It remains unclear whether HPV status is an independent predictor of feeding tube requirement, with one study confirming an increased risk [76] and another study finding no association [77], thus identifying further areas for future research. It is therefore imperative that nutrition support recommendations are continually reviewed in the context of evolving treatments and clinical practice.

## 5. Conclusions

In summary, our guidelines developed in 2010 are still able to predict with moderate to high accuracy those patients requiring proactive gastrostomy in the era of H-IMRT. The guidelines are recommended for aiding clinical decision-making in practice and informed consent discussions in consultation with patients to determine feeding tube preferences. Proactive tube placement should be viewed as a supportive intervention to enable patients to meet their nutrition requirements, minimize critical weight loss, complete radiotherapy without unplanned treatment break, complete optimal chemotherapy regimen, and improve short-term quality of life. To alleviate concerns with tube dependency, it is recommended that concurrent oral intake and swallowing rehabilitation programs should also be encouraged to optimize long-term swallowing outcomes and function. For patients not identified as requiring proactive gastrostomy tubes using the guidelines, they can still be managed through reactive nutrition support pathways, as outlined in the guidelines if required. Based on the results from this revalidation and logistic regression analysis of additional predictors, and in conjunction with the recent published literature, we have suggested a revision and update to the guidelines’ high-risk category definition to reduce the placement of unnecessary feeding tubes. The ongoing evaluation of the guidelines’ high-risk definition remains important as oncology treatments continue to evolve.

## Figures and Tables

**Table 1:** Comparison of patient demographics and clinical characteristics.

Patient Characteristics		Current Cohort 2013–2014 (N = 315)		Historical Cohort 2010–2011 (N = 270)		*p* Value
		N	Frequency (%)	N	Frequency (%)	
Age (mean ± SD)		65.13 ± 12.52 (Range 28–92)		63.15 ± 12.91 (Range 15–90)		0.061
Sex						0.741
	Male	239	76%	208	77%	
	Female	76	24%	62	23%	
Tumor Site						0.622
	Oral Cavity	84	27%	81	30%	
	Oropharynx	77	24%	65	24%	
	Nasopharynx	10	3%	4	1%	
	Hypopharynx	12	4%	14	5%	
	Larynx	30	10%	18	7%	
	Unknown Primary	11	3%	9	3%	
	Other	91	29%	79	29%	
T Classification ^1^						0.258
	T0	20	6%	13	5%	
	T1	56	18%	45	17%	
	T2	86	27%	69	26%	
	T3	47	15%	34	13%	
	T4	69	22%	60	22%	
	Tx	12	4%	9	3%	
	Recurrent	22	7%	38	14%	
	Other	3	1%	2	1%	
N Classification ^2^						0.006 *
	N0	126	40%	93	34%	
	N1	35	11%	45	17%	
	N2	125	40%	88	33%	
	N3	4	1%	4	1%	
	Recurrent	22	7%	38	14%	
	Other	3	1%	2	1%	
Treatment ^3, 4, 5^						0.582
	Surgery	59	19%	40	15%	
	RT	36	11%	28	10%	
	CRT	93	30%	91	34%	
	Surgery and PORT	104	33%	96	36%	
	Surgery and POCRT	20	6%	15	6%	
	Other	3	1%	0	0%	
Radiotherapy Details						<0.001 *
	H-IMRT	193	76%	75	33%	
	3D-conformal	62	24%	155	67%	
Chemotherapy details ^6^						0.216
	Cisplatin	91	78%	90	85%	
	Cetuximab	22	19%	16	15%	
	R-CHOP	3	3%	0	0%	

Abbreviations: SD, Standard Deviation; RT, radiotherapy; CRT, chemoradiotherapy; PORT, post-operative radiotherapy; POCRT, post-operative chemoradiotherapy; H-IMRT, helical intensity-modulated radiotherapy; R-CHOP, (Rituximab–Cyclophosphamide, Hydroxydaunorubicin, Oncovin, Prednisone). ^1^ Collapsed recurrent and other; ^2^ collapsed recurrent and other, and N2 with N3; ^3^ collapsed other with RT alone; ^4^ collapsed CRT with RT; ^5^ collapsed POCRT with PORT; ^6^ collapsed cetuximab with RCHOP. * *p* < 0.05 statistically significant.

**Table 2:** Diagnostic accuracy of the head and neck guidelines.

		Met Outcome Criteria for Primary Patient Outcome		Positive and Negative Predictive Values
		Yes * (Needed proactive gastrostomy) N = 101	No ^†^ (Did not need proactive gastrostomy) N = 214	
Met criteria for proactive gastrostomy as per guidelines	Yes ^‡^ N = 104	74 (TP)	30 (FP)	PPV = TP/(TP + FP) 71%
	No ^§^ N = 211	27 (FN)	184 (TN)	NPV = TN/(FN + TN) 87%
Sensitivity and specificity		Sensitivity = TP/(TP + FN) 73%	Specificity = TN/(FP + TN) 86%	

Abbreviations: TP, true positive; FP, false positive; FN, false negative; TN, true negative; PPV, positive predictive value; NPV, negative predictive value. * Positive prediction = met the predefined primary patient outcome “needed proactive gastrostomy”. The patient used a proactive or reactive feeding tube for >4 weeks, OR the patient did not use a proactive or reactive feeding tube for >4 weeks AND had >10% weight loss. ^†^ Negative prediction = did not meet the predefined patient outcome “did not need proactive gastrostomy”. Patient did not use a proactive or reactive feeding tube for >4 weeks AND had <10% weight loss. ^‡^ Recommended for proactive gastrostomy insertion as per guideline criteria. ^§^ Not recommended for proactive gastrostomy insertion as per guideline criteria.

**Table 3:** Change to high-risk category for predicting patients requiring a proactive gastrostomy and summary of guideline validation studies.

Development Date	Original (2007)	Updated (2010)	Revised (2021)
Clinical criteria	Oral + bilateral CRT Midline oropharynx + CRT Nasopharynx + CRT Pharynx + CRT	Oral + bilateral CRT Oropharynx + bilateral CRT Nasopharynx + CRT Hypopharynx + CRT UKP + CRT	Oral (T3 or T4) + CRT Oropharynx (T3 or T4) + CRT Nasopharynx (T3 or T4) + CRT All hypopharynx + CRT
Nutrition or swallowing criteria	Dysphagia at presentation or Severe malnutrition at presentation - Weight loss > 10% in 6 months - BMI < 18.5 - BMI < 20 + weight loss 5–10% in 6 months - SGA C - Poor oral intake (minimal for >5d and/or unlikely to improve for >5d)	Severe malnutrition at presentation - Weight loss > 10% in 6 months - BMI < 18.5 - BMI < 20 + weight loss 5–10% in 6 months - SGA C	Nil
Clinical criteria combined with nutrition or swallowing criteria	Nil	Nil	Oral (T1 or T2) + CRT Oropharynx (T1 or T2) + CRT Nasopharynx (T1 or T2) + CRT and baseline nutrition or swallowing risk factors - Weight loss >= 10% - Low BMI < 18.5 - Dysphagia
Validation results	**3D-conformal cohort (2007–2008)** [27] Sensitivity 54% Specificity 93% PPV 82% NPV 77%	**3D-conformal and H-IMRT cohort (2010–11)** [34] Sensitivity 72% Specificity 96% PPV 92% NPV 82% **H-IMRT cohort (2013–2014)** Sensitivity 73% Specificity 86% PPV 71% NPV 87%	Nil

Abbreviations: CRT, chemoradiotherapy; BMI, body mass index; SGA, subjective global assessment; H-IMRT, helical intensity-modulated radiotherapy; PPV, positive predictive value; NPV, negative predictive value.

**Table 4:** Patient characteristics of those pre-defined as high-risk as per guidelines but not meeting true indication for proactive gastrostomy (false positive cases n = 30).

Clinical Characteristic		N (%)
Treatment	Surgery Alone *	2 (7%)
	Chemoradiotherapy	25 (83%)
	Post-operative chemoradiotherapy	3 (10%)
Tumor Site	Oral Cavity	5 (17%)
	Oropharynx	20 (67%)
	Nasopharynx	1 (3%)
	Unknown Primary	4 (13%)
T Stage	T0/Tx	4 (13%)
	T1	4 (13%)
	T2	14 (47%)
	T3	4 (13%)
	T4	4 (13%)

* Classified as high-risk based on severe malnutrition at diagnosis.

**Table 5:** Univariate and multivariate analysis table for determining associations with the need for a proactive gastrostomy.

Characteristic	Univariate Analysis			Multivariable Analysis		
	OR	95% CI	*p*-Value	OR	95% CI	*p*-Value
Sex			0.081			NS
Male	RL					
Female	0.58	0.31–1.07				
Age			0.002			NS
Continuous variable	0.97	0.95–0.99				
Nutrition risk using MST			0.058			NS
Not at risk (0–1)	RL					
At risk (≥2)	1.67	0.98–2.84				
BMI			0.139			
Normal (18.5–25 kg/m^2^)	RL					
Underweight (<18.5 kg/m^2^)	0.68	0.23–1.99				
Overweight (>25 to 30 kg/m^2^)	1.50	0.82–2.75				
Obese (>30 kg/m^2^)	1.83	0.96–3.47				
Tumor site			<0.001			0.017
Oral cavity	RL			RL		
Oro-/nasopharynx	3.57	1.88–6.77		0.92	0.38–2.21	
Hypopharynx/larynx	0.90	0.38–2.08		0.38	0.13–1.11	
UKP/unable to ascertain/other	0.13	0.05–0.37		0.19	0.06–0.60	
Treatment modality			<0.001			<0.001
Surgery or RT alone	RL			RL		
PORT	2.35	0.91–6.02		2.38	0.84–6.73	
CRT or POCRT	22.8	9.59–54.62		8.86	3.07–25.57	
T stage			<0.001			0.004
T1	RL			RL		
T2	1.30	0.61–2.78		0.85	0.32–2.24	
T3/T4	2.70	1.33–5.48		2.62	1.04–6.63	
Tx/T0	0.20	0.04–0.95		0.16	0.02–1.57	
N stage			<0.001			NS
N0	RL					
N1	0.88	0.33–2.36				
N2–3	4.60	2.61–8.09				
Diet texture pre-treatment			0.031 *			
Full/soft	RL					
Minced/puree	2.13	1.07–4.21				

Abbreviations: OR, odds ratio; CI, confidence interval; RL, reference level; NS, not significant; MST, malnutrition screening tool; BMI, body mass index; UKP, unknown primary; RT, radiotherapy; PORT, post-operative radiotherapy; CRT, chemoradiotherapy; POCRT, post-operative chemoradiotherapy. * Not included in the multivariate model was the association with nutrition risk (MST) in the bivariate comparison. Note that when the diet texture was entered instead of MST as a variable in the model, it was still NS.

## Data Availability

Data supporting reported results can be requested via the corresponding author.
